# Case Report: Primary endodermal sinus tumor on the abdominal wall near the right liver: a diagnostic pitfall

**DOI:** 10.3389/fonc.2023.1185129

**Published:** 2023-10-18

**Authors:** Shuo Chen, Feng Chen, Xiao Xu

**Affiliations:** ^1^ Department of Radiology, First Affiliated Hospital, Zhejiang University School of Medicine, Hangzhou, Zhejiang, China; ^2^ Department of Radiology, Shaoxing People’s Hospital (Shaoxing Hospital, Zhejiang University School of Medicine), Shaoxing, Zhejiang, China; ^3^ Key Laboratory of Functional Molecular Imaging of Tumor and Interventional Diagnosis and Treatment of Shaoxing City, Shaoxing, Zhejiang, China

**Keywords:** endodermal sinus tumor, abdominal wall, ultrasonography, Doppler, color, tomography, X-ray computed, magnetic resonance imaging

## Abstract

**Background:**

Endodermal sinus tumors are rare, especially extragonadal endodermal sinus tumors, which often occur in the midline of the brain, neck, chest, and abdomen.

**Case summary:**

We present the case of a 37-year-old woman with a mass on the right edge of the liver. Color Doppler ultrasound, computed tomography, and magnetic resonance imaging examinations were performed before the operation. Given these results and the elevation of alpha-fetoprotein, the diagnosis of hepatocellular carcinoma was made. Postoperative pathological examination indicated an endodermal sinus tumor on the abdominal wall near the right liver. The causes of misdiagnosis were analyzed, and the related literature was reviewed.

**Conclusion:**

Primary endodermal sinus tumors on the abdominal wall near the right liver are easily misdiagnosed as hepatocellular carcinoma due to liver compression and elevated alpha-fetoprotein. The key point of differentiation is the wide basal connection between the tumor and the abdominal wall near the liver. In addition, the enhancement mode of endodermal sinus tumors is different from the enhancement pattern of hepatocellular carcinoma.

**Core tip:**

Extragonadal endodermal sinus tumors often occur in the midline of the body. Here, we present a case of a primary endodermal sinus tumor on the abdominal wall near the right liver for the first time.

## Introduction

An endodermal sinus tumor (EST; also known as a yolk sac tumor) is a primitive malignant germ cell tumor that is histologically similar to the mesenchyme of the primitive yolk sac (1, 2). It rarely arises from somatic cells (such as endometrioid epithelial tumors) through reverse differentiation. EST typically occurs in the gonads. The common sites of extragonadal EST are midline structures, including the mediastinum, retroperitoneum, and sacral region. Primary EST on the abdominal wall near the right liver has not been reported so far. We present a case of a primary EST on the abdominal wall near the right liver misdiagnosed as hepatocellular carcinoma (HCC) before surgery.

## Case presentation

### Chief complaints

Physical examination showed that alpha-fetoprotein increased over 3 months, and a liver mass was found 0.5 months earlier.

### History of present illness

Half a month ago, the patient developed persistent low back pain for 1 day without inducement.

### History of past illness

The patient had no personal or family history.

### Physical examination

There was no specific finding on physical examination.

### Laboratory examinations

Alpha-fetoprotein (AFP) was 206.2 ng/ml.

### Imaging examinations

**Figure 1:**
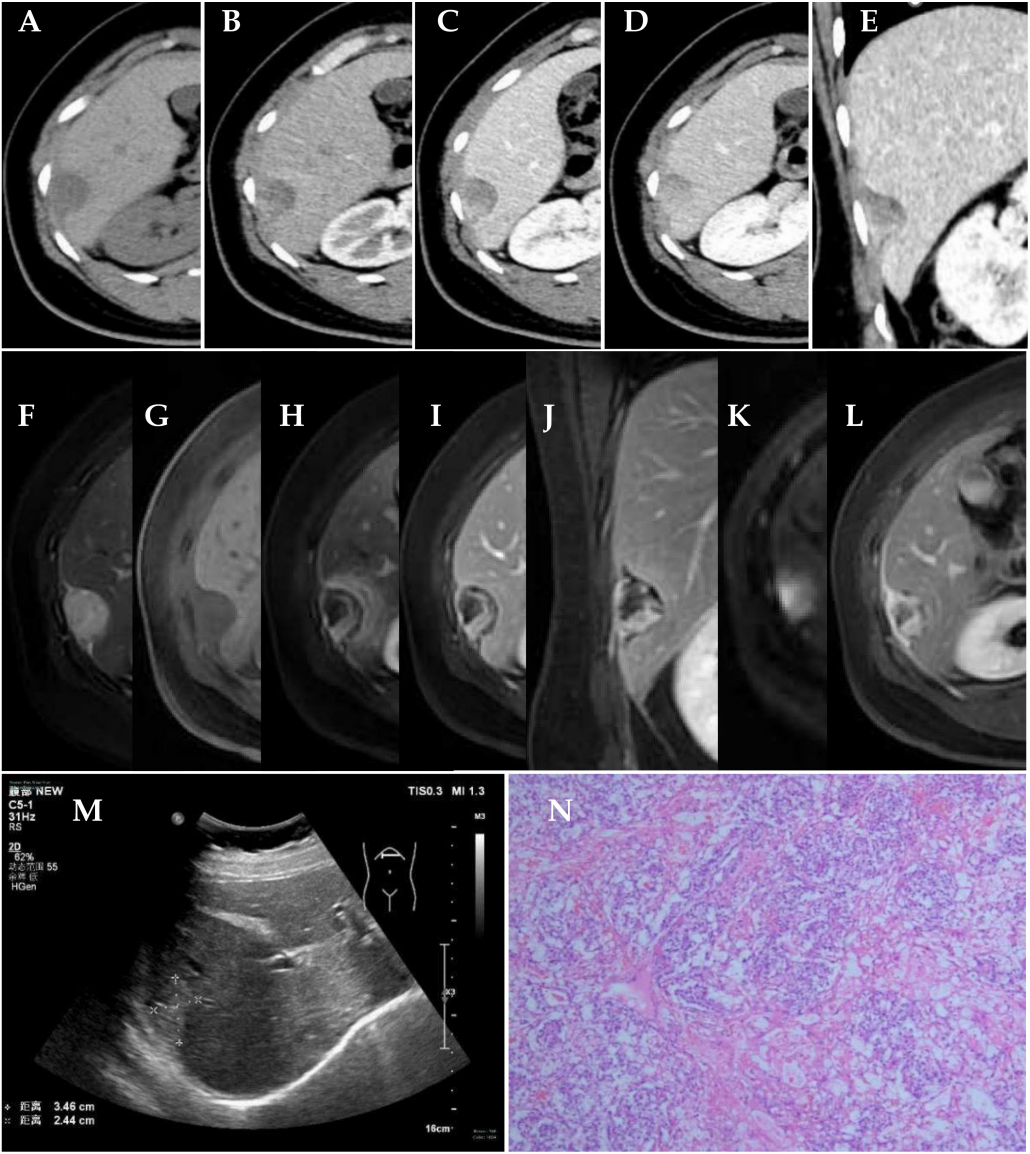
Clinical examination. **(A–E)** CT scan of the abdomen; axial preenhanced, axial arterial-phase, axial portal-phase, axial solid-phase, and coronal portal-phase views showed a tumor on the right edge of the liver. **(F–L)** MRI scan of abdomen. Axial T2WI FS, axial T1WI FS, axial arterial-phase, axial portal-phase, coronal parenchymal-phase, axial DWI, and axial delayed-phase views showed a tumor on the right edge of the liver. **(M)** Color Doppler ultrasound showed a tumor on the right edge of the liver. **(N)** Histology of the mass revealed Schiller–Duval bodies (×50).

Color Doppler ultrasound showed a strong echo with a range of 3.5 cm × 2.4 cm on the right edge of the liver. No obvious blood flow signal was found (**Figure 1M**).

The computed tomography (CT) scan showed a round, low-density mass on the right edge of the liver, with a size of approximately 3.1 cm × 1.9 cm. On contrast-enhanced images, non-enhanced liquefaction and necrotic zones were found in a cystic-solid mass. The solid part of the enhancement was located on the periphery of the mass, with irregular strips showing progressive and obvious enhancement. As time went on, the enhancement range of the mass expanded to the center. The CT value of the solid part was approximately 42 HU in the plain CT scan, approximately 90 HU in the arterial phase, approximately 111 HU in the portal vein phase, and approximately 107 HU in the solid phase (**Figures 1A–E**).

A magnetic resonance imaging (MRI) scan showed a round long T1 and long T2 signal mass on the right edge of the liver, which showed high signal intensity on diffusion-weighted imaging (DWI) (b = 1,000 s/mm^2^). The average apparent diffusion coefficient (ADC) value of the tumor was 2.24 × 10^3^ mm^2^/s. The size of the mass was approximately 3.0 cm × 2.1 cm. On contrast-enhanced images, non-enhanced liquefaction and necrotic zones were found in a cystic-solid mass. The solid part of the enhancement was located in the periphery of the mass, with irregular strips, showing progressive and obvious enhancement. The enhancement of the liver parenchyma around the mass in the arterial phase showed a halo sign. An enhanced capsule was seen on the surface of the mass (**Figures 1F–L**).

## Treatment

Surgery was performed. The tumor was located on the abdominal wall near the right liver. It squeezed the liver and had no obvious adhesion to the liver. It had a capsule and was approximately 3 cm in diameter. An EST on the abdominal wall near the right liver was confirmed by postoperative pathology. After the operation, the patient received four cycles of cisplatin, etoposide, and bleomycin (BEP) combined with chemotherapy.

## Pathology

Pathological examination indicated an EST on the abdominal wall near the right liver. Pathologically, cuboidal tumor cells were arranged in a microcystic structure. Their cell nuclei were large, deeply stained, or vacuolated, and nuclear division was active. Schiller–Duval bodies could be seen in the region. The tumor stroma was rich in blood vessels (**Figure 1N**). Immunohistochemically, the tumor cells were positive for CK (Pan), AFP, GPC-3, SALL4, and PLAP; weakly positive for PAX-8; partially positive for CDX2; and negative for Napsin A, CK7, Oct-4, and TTF-1.

## Outcome and follow-up

The patient was followed up for two years, and no recurrence was found.

## Discussion

EST is a rare tumor that can occur in both sexes and may be found in the ovary, testes, and other body parts. EST is commonly observed in young children. The cause of EST is essentially unknown. Some studies suggest that RUNX3 gene hypermethylation and GATA-4 overexpression may be involved in the pathogenesis of EST (3, 4). The Schiller–Duval body is pathognomonic for EST, and it appears like a glomerulus in structure with a fibrovascular core. In this case, the histology of the EST revealed Schiller–Duval bodies. This EST on the abdominal wall near the right liver was a primary tumor, and no tumor was found in other parts of the body.

Patients with EST have signs and symptoms that depend on the location of the tumor. This patient developed persistent low back pain for 1 day without inducement for 0.5 months before seeing the doctor in our hospital. The increase in alpha-fetoprotein is one of the important features of EST. In this case, alpha-fetoprotein increased before the operation and returned to normal after the operation.

EST shows up as an enhancing solid cystic mass on CT and MR studies (5–8). In this case, the boundary of the EST was relatively clear, and an enhanced capsule could be seen. It was confirmed that the tumor had a capsule during the operation. The EST was highly malignant and grew rapidly, and it was accompanied by liquefaction and necrosis. It showed a mixed solid cystic mass, and unenhanced liquefaction and necrotic areas were confirmed by postoperative pathology. The enhanced solid part of the EST was located on the periphery of the tumor and was irregularly striped. The enhancement type of the EST was progressive enhancement. As time went on, the enhancement range of the tumor expanded to the center. The CT value of the solid part was approximately 42 HU in the plain CT scan, approximately 90 HU in the arterial phase, approximately 111 HU in the portal vein phase, and approximately 107 HU in the solid phase (**Figures 1A–D**).

The diagnosis of EST depends on history, physical examination, imaging studies, and blood chemistry. We discussed and analyzed the reasons for the misdiagnosis in this case. The diagnostician, combining the imaging result with the elevation of alpha-fetoprotein, subjectively first considered whether it was liver cancer. Although there were signs of compression of the liver by the lesion, we have also seen similar signs in exogenous liver cancer, and endodermal sinus tumors located at the edge of the liver are extremely rare, leading to misdiagnosis. This patient was misdiagnosed with HCC located in S6 of the liver before the operation. Careful analysis after the operation found that the tumor was connected to the abdominal wall near the right liver with a wide base (**Figures 1E, G**), suggesting that the tumor was located on the abdominal wall near the right liver rather than in the liver. In addition, S6 of the liver was compressed and deformed. The enhancement type of the EST was progressive enhancement, different from that of HCC. The characteristic enhancement pattern of HCC is that the enhancement extent of the tumor is greater than that of the liver parenchyma in the arterial phase and lower in the portal and sinusoidal phases.

The current treatment for EST is surgery and chemotherapy. The combination of cisplatin, etoposide, and bleomycin has shown a good response in most patients (9, 10). Our patient underwent surgery. After the operation, she received four cycles of BEP combined with chemotherapy. During the follow-up at two years, there was no recurrence.

## Conclusion

To our knowledge, this is the first report of a primary EST on the abdominal wall near the right liver. It was easily misdiagnosed as HCC due to liver compression and elevated alpha-fetoprotein. The key points of differentiation are as follows. First, the wide basal connection between the tumor and the abdominal wall near the liver indicates that the tumor is located on the abdominal wall near the liver rather than in the liver. Second, the enhancement mode of EST is progressive enhancement, which is different from that of HCC. The characteristic enhancement pattern of HCC is that the enhancement extent of the tumor is greater than that of the liver parenchyma in the arterial phase and lower in the portal and sinusoidal phases.

## Data availability statement

The original contributions presented in the study are included in the article/supplementary material, further inquiries can be directed to the corresponding author/s.

## Ethics statement

The studies involving human participants were reviewed and approved by the Institutional Review Board of First Affiliated Hospital, Zhejiang University School of Medicine. The patients/participants provided their written informed consent to participate in this study. Written informed consent was obtained from the individual(s) for the publication of any potentially identifiable images or data included in this article.

## Author contributions

SC conceived and wrote the manuscript; FC acquired, analyzed, or interpreted the data for the work. XX revised it critically for important intellectual content and agreed to be accountable for all aspects of the work by ensuring that questions related to the accuracy or integrity of any part of the work are appropriately investigated and resolved. All authors contributed to the article and approved the submitted version.
